# Enhanced Immunogenicity and Affinity with A35R-Fc-Based Chimeric Protein Compared to MPXV A35R Protein

**DOI:** 10.3390/v17010116

**Published:** 2025-01-16

**Authors:** Shimeng Bai, Yanxin Cui, Qibin Liao, Hongyang Yi, Zhonghui Liao, Gengwei Zhang, Fenfang Wu, Hongzhou Lu

**Affiliations:** 1 Bio-Therapeutic Center, Shenzhen Clinical Research Center for Infectious Disease, State Key Discipline of Infectious Disease, Shenzhen Third People’s Hospital, Second Hospital Affiliated to Southern University of Science and Technology, Shenzhen 518112, China; sophia15354815841@163.com (S.B.); liaoqibin1991@sina.cn (Q.L.); yihy2018@mail.sustech.edu.cn (H.Y.); gengwei@szsdsrmyy10.wecom.work (G.Z.); 2 School of Public Health, Bengbu Medical University, Bengbu 233030, China; yanxxinc@outlook.com (Y.C.); liaozhonghui@stu.bbmc.edu.cn (Z.L.)

**Keywords:** mpox, mpox virus, MPXV, A35R protein, Fc fusion protein

## Abstract

The re-emergence of the mpox pandemic poses considerable challenges to human health and societal development. There is an urgent need for effective prevention and treatment strategies against the mpox virus (MPXV). In this study, we focused on the A35R protein and created a chimeric A35R-Fc protein by fusing the Fc region of IgG to its C-terminal. We then assessed its reactivity with A35R-specific antibodies and human convalescent plasma, as well as its immunogenicity. Our findings indicate that the A35R-Fc protein significantly enhances affinity to A35R antibodies compared to the commercially available A35R protein and exhibits considerable reactivity to human plasma. Additionally, mice immunized with A35R-Fc exhibited increased neutralizing antibody titers against the live MPXV. These results support the potential of Fc domain chimeric antigens as a strategy to enhance the efficacy of subunit vaccines targeting the MPXV.

## 1. Introduction

Mpox is a severe infectious disease caused by the mpox virus (MPXV), which can lead to severe or even fatal outcomes. The recent mpox outbreak is the largest recorded outbreak in non-endemic countries. From 2022 to 2024, the World Health Organization (WHO) declared the mpox outbreak a global health emergency twice, urging immediate global public health responses [1,2]. Therefore, developing new strategies against MPXV is imperative to ensure complete protection.

Vaccination is the most effective strategy for preventing and controlling MPXV spread. However, there is currently no vaccine specifically designed for MPXV. Previous studies have demonstrated that smallpox vaccines provide approximately 85% cross-protection against MPXV [3]. And the other two available vaccines—ACAM2000 and JYNNEOS—have been authorized for use in high-risk populations as post-exposure prophylaxis [4]. Although these vaccines have been shown to generate robust neutralizing antibodies and T cell responses in vaccinated individuals, they can also lead to some side effects and exhibit limited efficacy [4,5,6,7,8]. Additionally, a study reported that 4% of the participants had breakthrough infections after postexposure vaccination with JYNNEOS [9]. Therefore, there is a pressing need for the development of safer and more effective MPXV-specific vaccines to prevent an MPXV pandemic.

MPXV is a member of the orthopoxvirus genus within the Poxviridae family. Like other poxviruses, it has a complex life cycle that produces two infectious forms: extracellular enveloped virus (EEV) and intracellular mature virus (IMV). They are antigenically distinct from each other and interact with the cell surface differently. IMVs are believed to be involved in host-to-host spread, while EEVs are thought to be involved in cell-to-cell viral transmission. The EEV form expresses approximately 25 membrane proteins on the mature virion, some of which are widely used in vaccine design or antibody targets [10], and some are highlighted as potential targets for rapid detection [11]. Among these, A35R, a 23 kDa type II transmembrane protein and homolog of vaccinia virus A33, plays a critical role in facilitating the spread of viral particles from cell to cell [12]. It forms homodimers on the EEV membrane, with a membrane-proximal cysteine on its ectodomain [13,14,15,16]. As an important viral membrane protein, MPXV A35R has been evaluated for vaccine development [17] and used as a potential target for MPXV serological detection [18]. However, previous studies show that immunization with A35R protein or individual A35 mRNA induced weak neutralizing antibodies against MPXV or VACV [19,20], thus leading to less protection in vivo [21].

The Fc domain fusion approach has been widely established during the development of Fc-based protein drugs and vaccine design. It presents advantages in promoting the correct folding of proteins and uptake by antigen-presenting cells, increasing protein solubility and stability, extending half-life, and enhancing antigen immunogenicity [22,23,24,25]. Additionally, the Fc chimeric proteins can be easily purified through protein A/G affinity chromatography. For all these reasons, an Fc fusion strategy is a valid way to improve antigen immunogenicity and affinity.

Our study aims to establish an Fc-fused A35R protein to improve its affinity and immunogenicity in order to induce effective protection in vivo, and our findings suggest that A35R-Fc could be a promising strategy for developing the next generation of mpox vaccines.

## 2. Materials and Methods

### 2.1. Ethics Statement

Animal studies were approved by the Ethics Committees of Shenzhen Third People’s Hospital (approval number: 2024-007; approval date: 7 February 2024). All mice experiments were conducted in compliance with the recommendations in the Guide for the Care and Use of Laboratory Animals of the Ethics Committee of Shenzhen Third People’s Hospital. The procedures used for euthanasia of study animals followed tenets of the ARRIVE reporting guidelines.

### 2.2. Cells and Viruses

CHO-K1 cells (ATCC, CCL-61) were cultured in Dulbecco’s modified Eagle medium/F-12 (DMEM/F-12, Gibco, Grand Island, NY, USA, Cat. #11320033). Vero-E6 (ATCC, CRL-1586) and HEK293T (ATCC, CRL-3519) cells were maintained in Dulbecco’s modified Eagle medium (DMEM, Gibco, Grand Island, NY, USA). All cultures were supplemented with 10% (*v*/*v*) fetal bovine serum and 1% (*v*/*v*) penicillin–streptomycin. The MPXV strain (hMpxV/China/SZ-SZTH42/2023 GISAID accession ID EPI_ISL_18213374) was isolated from the first mpox case in Shenzhen, China, as previously described [26].

### 2.3. Construction, Expression, and Purification of the A35R-Fc Fusion Protein

The A35R-Fc construct was generated by genetically fusing the MPXV A35R (GenBank MT350282.1; Arg58 to Thr181) at its C terminus with the human IgG1 heavy chain. The resulting plasmid constructs were transfected into CHO-K1 or HEK293T cells using PEIpro^®^ (Polyplus, Illkirch, Franch, Cat. #101000017) at a 1:3 DNA/PEI ratio. Supernatants were collected 4 days post-transfection and purified using Protein A/G 4FF resin (Sangon Biotech, Shanghai, China, Cat. #C600981). Briefly, the supernatant was gently applied to the resin at a controlled flow rate of 1 mL/min. The resin was then washed with a binding buffer equal to 10-fold column volume, followed by elution with an acidic buffer (pH 2–3) at 2-fold column volume. The outflowing liquid contained A35R-Fc protein. The purity of the recombinant proteins was assessed by 12.5% SDS-PAGE and visualized with Coomassie blue staining.

To detect A35R-Fc fusion proteins, cell culture supernatants were transferred onto a polyvinylidene fluoride (PVDF) membrane (Merck Millipore, Billerica, MA, USA, Cat. #IPFL00010) following SDS-PAGE, with the transfer performed for 1 h at 400 mA. The membrane was incubated with a primary antibody against A35R (Sino Biological, Beijing, China, Cat. #40886-T62) diluted 1:2000 to identify the A35R-Fc fusion protein. Subsequently, peroxidase-conjugated goat anti-rabbit immunoglobulin G (IgG) H&L (ZSGB-BIO, Beijing, China, Cat. #ZB-5305) was added at a dilution of 1:5000. Finally, tetramethylbenzidine (TMB) substrate (Sangon Biotech, Shanghai, China, Cat. #E661007) was applied for signal development.

### 2.4. Mouse Experiments

Female BALB/c mice (n = 5 per group, 6–8 weeks old) were purchased from Guangdong GemPharmatech and housed in a specific pathogen-free (SPF) environment. The mice were intramuscularly immunized twice, 14 days apart, with equimolar amounts of either 5 μg of commercial A35R (Antibody system, Franch, Cat. #EVV13101, expression system: mammalian cells) or 15 μg of A35R-Fc protein, each combined with 10 μg of CPG1018 and 100 μg of alum adjuvant. Mice immunized with PBS mixed with equal amounts of CPG1018 and alum adjuvant served as controls. Blood samples were collected on days 0, 14, 28, and 42 post-initial immunization to assess serum A35R-specific antibody levels.

### 2.5. Enzyme-Linked Immunosorbent Assay (ELISA)

To detect A35R-specific antibodies in mouse serum, ELISA plates (Corning, NY, USA, Cat. #3590) were precoated with 100 ng of A35R protein (Antibody system, Cat. #EVV13101) per well. Serially diluted mouse sera were added and incubated at room temperature (RT) for 2 h. An HRP-conjugated goat anti-mouse IgG antibody (Invitrogen, Carlsbad, CA, USA, Cat. #PA1-84388) was then added at a dilution of 1:5000 and incubated for 1 h at RT. Absorbance was measured at 450 nm using a Synergy microplate reader (Thermo Fisher Scientific, Waltham, MA, USA, Cat. #51119080). Endpoint titers were determined at the highest dilution, with the cutoff value defined as an OD value twice that of the control group.

To evaluate the reactivity of the A35R-Fc fusion protein, ELISA plates were coated overnight at 4 °C with 100 ng/well of A35R-Fc, using the commercial A35R protein (Antibody system, Franch, Cat. #EVV13101) as a positive control. Various commercial antibodies (Sino Biological, Beijing, China, Cat. #40886-T62; Antibody System, Franch, Cat. #RVV13101) were diluted twofold, starting at 0.5 μg/mL, and added to the wells for incubation. Following this, either HRP-conjugated goat anti-mouse (Invitrogen, Carlsbad, CA, USA, Cat. #PA1-84388) or HRP-conjugated goat anti-rabbit antibodies (Invitrogen, Carlsbad, CA, USA, Cat. #31460) were added, followed by signal development using the TMB Chromogen Solution (for ELISA) (Sangon Biotech, Shanghai, China, Cat. #E661007).

### 2.6. Surface Plasmon Resonance

The binding kinetics and affinity of antibodies to the proteins A35R, A35R-Fc, and A35R-Fc (CHO) were assessed using the Biacore 8K system (GE Healthcare, Buckinghamshire, UK). One flow cell of the CM5 Series S sensor chip (Cytiva, Wales, UK, Cat. #29149604) was covalently coated with each protein in 10 mM sodium acetate buffer (pH 4.5) to achieve a final response unit (RU) of approximately 200, and the other flow was left uncoated and blocked as a control. The protein concentrations used for coating were 1.75 μg/mL for A35R, 1.5 μg/mL for A35R-Fc, and 3 μg/mL for A35R-Fc (CHO). All assays were conducted at a flow rate of 30 µL/min in HBS-EP buffer (Cytiva, Wales, UK, Cat. #BR100669). Serially diluted antibodies were injected for 60 s, and the resulting data were analyzed using a 1:1 binding model with Biacore Evaluation software (GE Healthcare, Buckinghamshire, UK, version 3.0.12.15655). Each measurement was performed in duplicate, and the mean affinity constant and standard deviation were calculated.

SPR was also conducted for reactivity analysis with human plasma samples. The flow cells of the CM5 sensor chip were covalently coated with proteins to achieve a final RU of around 2000. Concentrations used for A35R, A35R-Fc, and A35R-Fc (CHO) were 10 μg/mL, 8.5 μg/mL, and 15 μg/mL, respectively. The same diluted plasma samples were injected for 60 s, and the maximum binding RU value at the end of the injection was recorded for analysis. Each measurement was duplicated, providing a mean RU.

### 2.7. MPXV Neutralization Assay

Sera were inactivated at 56 °C for 30 min to evaluate MPXV neutralization. The focus reduction neutralization test (FRNT) was performed as previously reported [27]. Serial dilutions of the serum samples were mixed with 200 focus-forming units (FFUs) of MPXV in 96-well microwell plates and incubated at 37 °C for 1 h in the presence of 10% rabbit complement (Cedarlane, Burlington, ON, Canada, Cat. #CL3441-S100-R). The mixtures were then transferred to 96-well plates seeded with Vero E6 cells and incubated at 37 °C for 18 h. After incubation, the supernatant was removed, and the cells were fixed with 4% paraformaldehyde for 30 min. The cells were then permeabilized with 0.2% Triton X-100 for 10 min and incubated with HRP-conjugated vaccinia virus polyclonal antibody (Invitrogen, Carlsbad, CA, USA, Cat. #PA1-73192) for 2 h at room temperature. The reactions were developed using KPL TrueBlue Peroxidase substrates (Seracare Life Sciences, Milford, MA, USA, Cat. #5510-0030). MPXV foci were quantified with an EliSpot reader, and neutralizing antibody titers were calculated as the 50% inhibitory dose (ID_50_), expressed as the serum dilution that achieved a 50% reduction in MPXV foci compared to the virus control. The MPXV FRNT assay was conducted in a biosafety level 3 (BSL-3) facility at the Shenzhen Third People’s Hospital.

### 2.8. Statistical Analysis

Prism 9.4 (GraphPad, San Diego, CA, USA) was used for all the analyses. A one-way ANOVA was conducted for multiple comparisons, followed by Tukey’s multiple comparison post-test for the comparison of each group. *p* < 0.05 was considered statistically significant.

## 3. Results

### 3.1. Construction and Production of Recombinant A35R-Fc Fusion Protein

**Figure 1:**
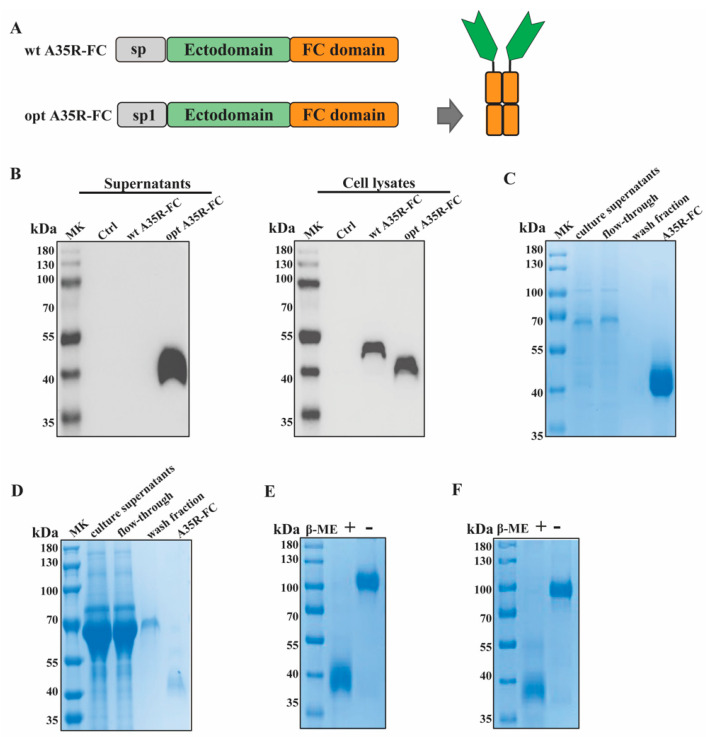
Characterization of A35R-Fc chimeric protein. (**A**) Diagrams for the design of Fc-based recombinant A35R proteins: the signal peptide was colored gray, the ectodomain was colored green, and the human IgG1 Fc domain was shown orange. The sp represents the sequence from the native A35R protein, and sp1 represents the sequence from the tissue plasminogen activator (tPA) signal peptide, so the chimeric A35R protein was shown as wt A35R-Fc or A35R-Fc, respectively. (**B**) Western blot for supernatants and cell lysate of wt A35R-Fc or A35R-Fc protein expressed in HEK 293T cells: The A35R protein band was detected by anti-A35R-specific antibody. Ctrl referred to negative control (DMEM medium). (**C**,**D**) SDS-PAGE analysis of protein A/G-purified forms of recombinant A35R-Fc expressed in HEK 293T (**C**) or CHO cells (**D**). (**E**,**F**) SDS-PAGE of A35R-Fc protein under reducing (+β-ME) and non-reducing conditions (−β-ME). Proteins from HEK 293F and CHO cells are shown in (**E**) or (**F**), respectively.

To obtain secreted recombinant A35R-Fc protein, we designed a fusion A35R protein (including the ectodomain, from R58 to T181) C-terminally attached to the Fc domain of the human IgG1 heavy chain, named as wt A35R-Fc (Figure 1A). Then expressed it by transient transfection of HEK 293T cells. Unfortunately, Western blot detected no signal in the culture supernatants using A35R rabbit antibody (Figure 1B). Then we tried to optimize it; the native signal peptide of the A35R protein was replaced with a tissue plasminogen activator (tPA) signal peptide (MDAMKRGLCCVLLLCGAVFVSPSQEIHARFRRGARA), creating A35R-Fc (Figure 1A). tPA is a commonly used heterologous signal peptide for increasing expression levels of recombinant proteins in mammalian hosts. The culture supernatants and cell lysates were collected on day 4, and the expression levels were examined (Figure 1B). Surprisingly, high levels of A35R-Fc were detected in culture supernatants and expected-sized bands were seen in the cell lysates. Our findings provided a more effective approach for the secretion of recombinant A35R-Fc protein.

For future functional analysis, different mammalian cell lines were selected for the recombinant A35R-Fc protein. As shown in Figure 1C,D, human HEK293T cells or CHO-K1 cells were side-by-side transfected with A35R-Fc plasmids, and culture supernatants were collected on Day 4. Affinity chromatography was performed to purify the fusion A35R-Fc proteins. The culture supernatant was diluted with binding/wash buffer (Lane 1) and applied to a gravity column containing Protein A/G resin. The flow-through was collected (Lane 2), and the column was washed twice with binding/wash buffer (Lane 3). Bound proteins were then eluted with elution buffer containing 0.1 M glycine (Lane 4) and neutralized with 1 M Tris-HCl. A prominent band around 40 kDa was observed in the elution fractions (Figure 1C,D). The target proteins produced in HEK293T cells were named A35-Fc, while those from CHO-K1 cells were called A35R-Fc (CHO).

Then, we characterized the proteins using SDS-PAGE under both reducing and nonreducing conditions (Figure 1E,F). As shown in Figure 1E, a band around 40 kDa under reducing conditions and a single band of approximately 100 kDa under nonreducing conditions were detected in polyacrylamide gels. The A35R-Fc protein expressed by CHO-K1 cells exhibited similar sizes (Figure 1F). Overall, the protein analysis shows that the A35R domains dimerized through the Fc domain.

### 3.2. A35R-Fc Chimeric Protein Showed High Binding Affinity to Different A35R-Specific Antibodies

**Figure 2:**
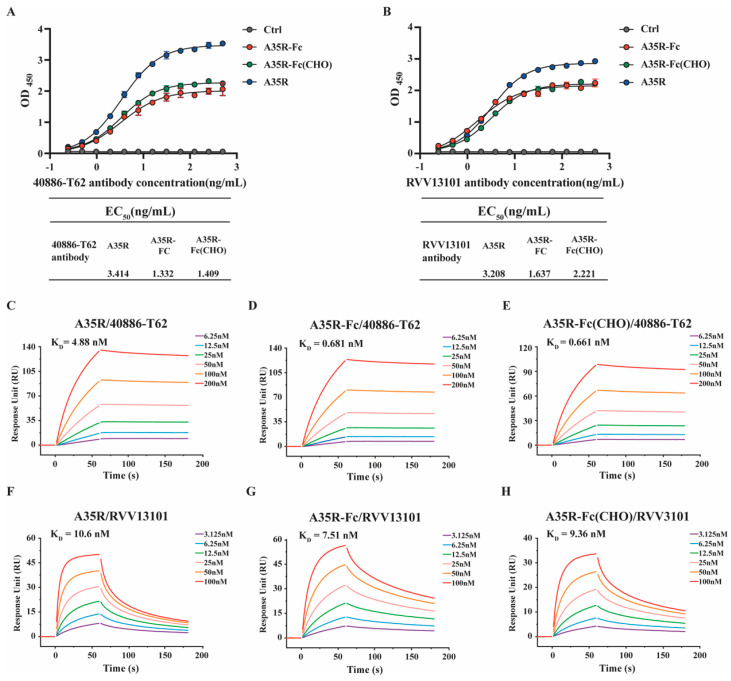
Characterization of affinity and binding kinetics analysis for A35R-Fc chimeric protein with different A35R-specific antibodies. (**A**,**B**) Binding of the A35R-Fc chimeric protein to antibody 40886-T62 (**A**) or RVV13101 (**B**) by enzyme-linked immunosorbent assay (ELISA). Ctrl represented negative control (BSA). The commercial A35R protein is the positive control. (**C**–**H**) Characterization of the affinity of A35R, A35R-Fc, or A35R-Fc (CHO) with antibody 40886-T62 (**C**–**E**) or RVV13101 (**F**–**H**) using SPR. The actual responses (colored lines) are shown. The equilibrium dissociation constant KD was calculated as kd/ka. The EC_50_ was calculated by fitting the OD_450_ values from serially diluted antibody 40886-T62 or RVV13101 to a sigmoidal dose–response curve.

To assess the proper activity of the A35R-Fc fusion proteins, we used ELISA and SPR to characterize the affinity and binding kinetics to antibodies 40886-T62 or RVV13101. ELISA showed that both antibodies 40886-T62 and RVV13101 could detect the commercial A35R or A35R-Fc fusion proteins. A35R-Fc or A35R-Fc (CHO) retained a strong binding activity to antibody 40886-T62 (EC_50_ values of 1.332 or 1.409 ng/mL, respectively) (Figure 2A), while the binding of A35R to 40886-T62 or RVV13101 antibodies was reduced, with an EC_50_ value of 3.414 or 3.208 ng/mL (Figure 2A,B). SPR was further performed to determine the binding affinity of Fc fusion proteins. It showed that the affinity of the antibody 40886-T62 for the chimeric A35R-Fc increased by about 8-fold compared with the commercial A35R protein, with K_D_ values of 0.681 nM (A35R-Fc) and 0.661 nM (A35R-Fc (CHO)) (Figure 2C–E) (Table 1). The affinity of the antibody RVV13101 for the chimeric A35R-Fc was also improved compared with the A35R protein (Figure 2F–H) (Table 1).

Overall, the affinity of Fc fusion proteins against antibodies was markedly enhanced compared to that of the commercial A35R protein, which was likely attributed to the dimerization of the Fc fragment.

### 3.3. A35R-Fc Chimeric Protein Could Be Used for Serological Assays of MPXV Infection

**Figure 3:**
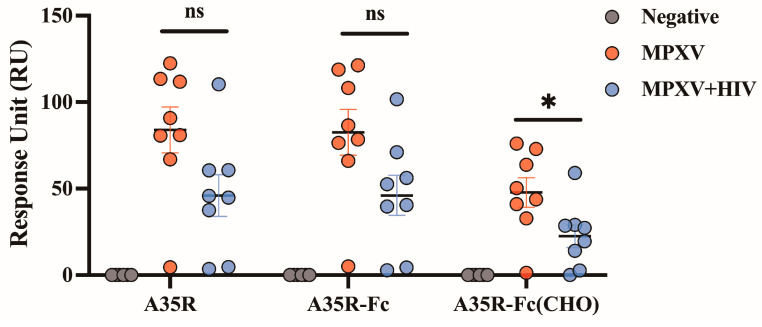
Binding ability evaluation of A35R-Fc for human plasma samples. * *p*-value < 0.05; ns: not significant.

To further evaluate the reactivity of the chimeric protein and its potential for detecting MPXV infection, we assessed the binding activity of A35R-Fc with human plasma samples obtained from patients recovered from MPXV-infected or co-infected with MPXV and HIV, using surface plasmon resonance. The results showed that the chimeric protein exhibited significant reactivity with most convalescent plasma samples (both MPXV and MPXV-HIV), demonstrating RU values of 73.86 for A35R-Fc and 46.11 for A35R-Fc (CHO) (Figure 3, Table 2), which were comparable to those of the commercial A35R protein. Importantly, no binding was detected with six individual negative control plasma samples (Figure 3 and Figure S1). It is worth noting that the binding RU values of A35R-Fc with convalescent plasma derived from patients infected with MPXV only are significantly higher than those co-infected with MPXV and HIV (mean RU values of 73.86 for MPXV infection and 46.11 for MPXV and HIV co-infection), which is consistent with previous reports that indicate the HIV infection weakens the antibody response against MPXV [28]. These results suggest that our recombinant A35R-FC could serve as an effective antigen for the identification of the MPXV infection.

### 3.4. A35R-Fc Protein Potently Enhances the Induction of Neutralizing Antibodies than A35R Protein in the Presence of Alum Plus CPG Adjuvants

**Figure 4:**
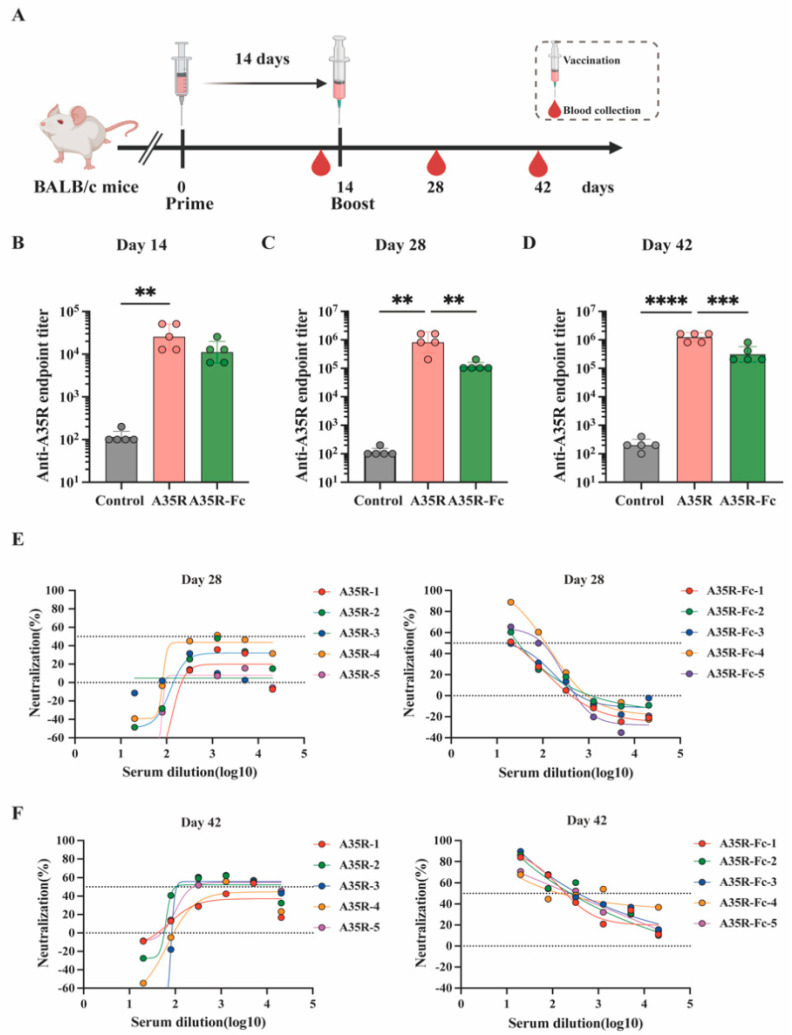
Immunization with the chimeric A35R-Fc enhances the induction of MPXV-neutralizing antibodies in mice. (**A**). Schematic diagram of immunization and sample collection. BALB/c mice (n = 5/group) were intramuscularly immunized twice with 5 μg of A35R or 15 μg of the Fc-fused A35R, formulated with CPG1018 or alum adjuvant with a 14-day interval. A group of mice was injected with empty-LNP as a control group. After immunization, the serum was collected at point times. Specific antibodies against A35R in the serum were tested by an enzyme-linked immunosorbent assay at day 14 (**B**), day 28 (**C**), and day 42 (**D**). Neutralizing antibody titers against MPXV at day 28 (**E**), day 42 (**F**) were detected by a FRNT assay. Titer data are expressed as the GMT ± SD. One-way ANOVA followed by Tukey’s multiple comparisons tests was performed for all comparisons. ** *p*-value < 0.01; *** *p*-value < 0.001; **** *p*-value < 0.0001.

To determine the immunogenicity of Fc fusion proteins in vivo, BALB/c mice received two intramuscular injections with equimolar amounts of the A35R-Fc or non-fused A35R in the presence of CPG1018 or alum as adjuvants (Figure 4A). ELISA tested the specific anti-A35R antibody titers. Notably, mice injected with both A35R-Fc or A35R exhibited detectable titers (GMT over 10^3^) of anti-A35R IgG after the first immunization and reached maximal titers (GMT over 10^6^) following the second dose (Figure 4B). Serum titers from mice immunized with the A35R protein were significantly higher (*p* < 0.01) than those from the group vaccinated with A35R-Fc at day 28 and 42 post-immunization (Figure 4C,D), which may be attributed to the use of A35R as the coated protein in the ELISA. To further validate the virus neutralization effects induced by the Fc fusion proteins, we also determined whether the serum could inhibit the infectivity of MPXV. Surprisingly, as shown in Figure 4E,F, only sera collected from mice immunized with A35R-FC were capable of efficiently neutralizing live MPXV, with an average ID50 of 236.9 four weeks after the second dose.

Overall, these data indicate that the virus neutralization effects of A35R were notably enhanced by fusion with an Fc domain, which provides a potential approach for designing subunit vaccines against MPXV.

## 4. Discussion

Since early May 2022, the human mpox outbreak has re-emerged, spreading to numerous countries.

The lethal Clade I MPXV outbreak in the Democratic Republic of Congo led to a dramatic increase in cases in 2024. Therefore, it is imperative to develop strategies to control this disease pandemic.

VACV A33R, the ortholog of MPXV A35R, has been identified as a target of immunotherapeutics or as a protective target of subunit vaccines, either alone or in combination with other membrane proteins [29,30]. Although A33R and A35R share significant homology, their differences may influence the cross-protection of smallpox vaccines against MPXV. For instance, the monoclonal antibody 1G10 binds strongly to VACV A33R but fails to recognize A35R [31]. Additionally, studies indicate that vaccination with A35R elicits low titers of neutralizing antibodies and exhibits weak immunogenicity [19,20]. Thus, enhancing the immunogenicity of A35R is crucial for improving its recognition by the host immune system, given its potential as a novel therapeutic or antiviral candidate.

In this study, we genetically fused the human IgG1 antibody Fc fragment to A35R, thus constructing the fusion protein A35R-Fc. The recombinant A35R-Fc fusion protein was produced using the CHO-K1 mammalian or human HEK293T cell expression system to enhance immunogenicity and ensure proper folding and glycosylation. We then compared the reactivity of two A35R-specific antibodies with commercial A35R protein and A35R-Fc fusion proteins derived from two different expression systems. Results showed that antibody 40886-T62 exhibited higher reactivity to both A35R-Fc and A35R-Fc (CHO) proteins compared to the commercial A35R protein, while the RVV13101 antibody displayed slightly higher reactivity as well. We also assessed the ability of these proteins to detect antibody levels in convalescent plasma samples from individuals infected with MPXV or co-infected with MPXV and HIV, demonstrating their potential as diagnostic agents.

Furthermore, we investigated the immunological characteristics of the A35R-Fc fusion protein using a two-dose immunization regimen. Although our results indicated that the commercial A35R protein elicited higher antibody titers than the chimeric A35R-Fc, it was notable that the commercial protein resulted in lower MPXV neutralizing activity. This discrepancy may be attributed to the fact that mice immunized with A35R-FC may induce more potent Fc effector functions against the extracellular enveloped virus (EEV) [32]. Specifically, the chimeric A35R-FC protein might enhance the activation of antibody-dependent mechanisms, such as Fc receptor (FcR) engagement and antibody-dependent cellular cytotoxicity (ADCC) [21,33], which need further research, such as in vivo challenge experiments to assess the effectiveness of the A35R-Fc protein.

Collectively, our results highlight the potential of Fc domain fusion as a promising approach to enhance the immunogenicity of the A35R protein. This strategy may represent a novel method for effectively controlling the MPXV pandemic.

## Figures and Tables

**Table 1:** Affinity and binding kinetics of A35R-Fc chimeric protein with 40886-T62 or RVV13101 antibody determined by surface plasmon resonance.

	K_D_ (M)	K_a_ (1/Ms)	K_d_ (1/s)	ꭓ^2^
A35R				
40886-T62	4.88 × 10^−9^	1.66 × 10^−5^	8.10 × 10^−4^	1.890
RVV13101	1.06 × 10^−8^	5.81 × 10^−6^	6.15 × 10^−2^	3.150
A35R-Fc				
40886-T62	6.81 × 10^−10^	1.25 × 10^−5^	8.54 × 10^−5^	0.949
RVV13101	7.51 × 10^−9^	9.91 × 10^−5^	7.45 × 10^−3^	2.140
A35R-Fc (CHO)				
40886-T62	6.61 × 10^−10^	1.62 × 10^−5^	1.07 × 10^−4^	1.410
RVV13101	9.36 × 10^−9^	2.01 × 10^−6^	1.88 × 10^−2^	1.210

**Table 2:** Binding ability of A35R-Fc chimeric protein with human convalescent plasma samples.

	RU (Mean, ±SD)
A35R	
MPXV	74.61, 44.79
MPXV + HIV	46.01, 34.13
Negative	0, 0
A35R-Fc	
MPXV	73.86, 43.74
MPXV + HIV	46.11, 32.81
Negative	0, 0
A35R-Fc (CHO)	
MPXV	42.45, 27.74
MPXV + HIV	22.52, 18.58
Negative	0, 0

## Data Availability

The data supporting the findings of this study are accessible within the main text and the Supplementary Information Files of the paper.

## Supplementary Materials

The following supporting information can be downloaded at https://www.mdpi.com/article/10.3390/v17010116/s1: Figure S1: Binding curves of A35R-FC with the human plasma samples determined by a biolayer interferometry assay.

## References

[1] Okwor T., Mbala P.K., Evans D.H., Kindrachuk J. (2023). A contemporary review of clade-specific virological differences in monkeypox viruses. Clin. Microbiol. Infect..

[2] World Health Organization 2022–24 Mpox (Monkeypox) Outbreak: Global Trends. https://worldhealthorg.shinyapps.io/mpx_global/.

[3] Isidro J., Borges V., Pinto M., Sobral D., Santos J.D., Nunes A., Mixao V., Ferreira R., Santos D., Duarte S. (2022). Phylogenomic characterization and signs of microevolution in the 2022 multi-country outbreak of monkeypox virus. Nat. Med..

[4] Rao A.K., Petersen B.W., Whitehill F., Razeq J.H., Isaacs S.N., Merchlinsky M.J., Campos-Outcalt D., Morgan R.L., Damon I., Sánchez P.J. (2022). Use of JYNNEOS (Smallpox and Monkeypox Vaccine, Live, Nonreplicating) for Preexposure Vaccination of Persons at Risk for Occupational Exposure to Orthopoxviruses: Recommendations of the Advisory Committee on Immunization Practices—United States, 2022. MMWR Morb. Mortal. Wkly. Rep..

[5] Xiang Y., White A. (2022). Monkeypox virus emerges from the shadow of its more infamous cousin: Family biology matters. Emerg. Microbes Infect..

[6] Lee J., Kwon S.L., Park J., Bae H., Lee H., Kwon G.Y. (2023). JYNNEOS vaccine safety monitoring in the Republic of Korea, 2022: A cross-sectional study. Osong Public Health Res. Perspect.

[7] Deng L., Lopez L.K., Glover C., Cashman P., Reynolds R., Macartney K., Wood N. (2023). Short-term Adverse Events Following Immunization With Modified Vaccinia Ankara-Bavarian Nordic (MVA-BN) Vaccine for Mpox. JAMA.

[8] Lim S.Y., Jung Y.M., Kim Y., Kim G., Jeon J., Chin B., Kim M.K. (2024). Adverse Reactions After Intradermal Vaccination With JYNNEOS for Mpox in Korea. J. Korean Med. Sci..

[9] Thy M., Peiffer-Smadja N., Mailhe M., Kramer L., Ferre V.M., Houhou N., Tarhini H., Bertin C., Beaumont A.L., Gare M. (2022). Breakthrough Infections after Postexposure Vaccination against Mpox. N. Engl. J. Med..

[10] Moss B. (2011). Smallpox vaccines: Targets of protective immunity. Immunol. Rev..

[11] Sagdat K., Batyrkhan A., Kanayeva D. (2024). Exploring monkeypox virus proteins and rapid detection techniques. Front. Cell. Infect. Microbiol..

[12] Perdiguero B., Blasco R. (2006). Interaction between vaccinia virus extracellular virus envelope A33 and B5 glycoproteins. J. Virol..

[13] Roper R.L., Payne L.G., Moss B. (1996). Extracellular vaccinia virus envelope glycoprotein encoded by the A33R gene. J. Virol..

[14] Duncan S.A., Smith G.L. (1992). Identification and characterization of an extracellular envelope glycoprotein affecting vaccinia virus egress. J. Virol..

[15] Matho M.H., Schlossman A., Meng X., Benhnia M.R., Kaever T., Buller M., Doronin K., Parker S., Peters B., Crotty S. (2015). Structural and Functional Characterization of Anti-A33 Antibodies Reveal a Potent Cross-Species Orthopoxviruses Neutralizer. PLoS Pathog..

[16] Su H.P., Singh K., Gittis A.G., Garboczi D.N. (2010). The structure of the poxvirus A33 protein reveals a dimer of unique C-type lectin-like domains. J. Virol..

[17] Fang Z., Monteiro V.S., Renauer P.A., Shang X., Suzuki K., Ling X., Bai M., Xiang Y., Levchenko A., Booth C.J. (2023). Polyvalent mRNA vaccination elicited potent immune response to monkeypox virus surface antigens. Cell Res..

[18] Yefet R., Friedel N., Tamir H., Polonsky K., Mor M., Hagin D., Sprecher E., Israely T., Freund N.T. (2022). A35R and H3L are Serological and B Cell Markers for Monkeypox Infection. medRxiv.

[19] Song S., Ren Z., Chen J., Li M., Jiang Y., Liu Y., Zhang B., Lu H., Zhao W., Shen C. (2024). Analysis of binding and authentic virus-neutralizing activities of immune sera induced by various monkeypox virus antigens. Immunol. Res..

[20] Galmiche M.C., Goenaga J., Wittek R., Rindisbacher L. (1999). Neutralizing and protective antibodies directed against vaccinia virus envelope antigens. Virology.

[21] Freyn A.W., Atyeo C., Earl P.L., Americo J.L., Chuang G.Y., Natarajan H., Frey T.R., Gall J.G., Moliva J.I., Hunegnaw R. (2023). An mpox virus mRNA-lipid nanoparticle vaccine confers protection against lethal orthopoxviral challenge. Sci. Transl. Med..

[22] Kontermann R.E. (2011). Strategies for extended serum half-life of protein therapeutics. Curr. Opin. Biotechnol..

[23] Mekhaiel D.N., Czajkowsky D.M., Andersen J.T., Shi J., El-Faham M., Doenhoff M., McIntosh R.S., Sandlie I., He J., Hu J. (2011). Polymeric human Fc-fusion proteins with modified effector functions. Sci. Rep..

[24] Roopenian D.C., Akilesh S. (2007). FcRn: The neonatal Fc receptor comes of age. Nat. Rev. Immunol..

[25] Loureiro S., Ren J., Phapugrangkul P., Colaco C.A., Bailey C.R., Shelton H., Molesti E., Temperton N.J., Barclay W.S., Jones I.M. (2011). Adjuvant-free immunization with hemagglutinin-Fc fusion proteins as an approach to influenza vaccines. J. Virol..

[26] Cheng L., Yang L., Wang M., Peng Y., Wang H., Yang X., Zhao J., Zhang M., Wang F., Zhang Z. (2024). Isolation and characterization of mpox virus from the first mpox case in Shenzhen, China. Virol. Sin..

[27] Ju B., Zhang Q., Ge J., Wang R., Sun J., Ge X., Yu J., Shan S., Zhou B., Song S. (2020). Human neutralizing antibodies elicited by SARS-CoV-2 infection. Nature.

[28] Moraes-Cardoso I., Benet S., Carabelli J., Perez-Zsolt D., Mendoza A., Rivero A., Alemany A., Descalzo V., Alarcón-Soto Y., Grifoni A. (2024). Immune responses associated with mpox viral clearance in men with and without HIV in Spain: A multisite, observational, prospective cohort study. Lancet Microbe.

[29] Chen Z., Earl P., Americo J., Damon I., Smith S.K., Yu F., Sebrell A., Emerson S., Cohen G., Eisenberg R.J. (2007). Characterization of chimpanzee/human monoclonal antibodies to vaccinia virus A33 glycoprotein and its variola virus homolog in vitro and in a vaccinia virus mouse protection model. J. Virol..

[30] Lustig S., Fogg C., Whitbeck J.C., Eisenberg R.J., Cohen G.H., Moss B. (2005). Combinations of polyclonal or monoclonal antibodies to proteins of the outer membranes of the two infectious forms of vaccinia virus protect mice against a lethal respiratory challenge. J. Virol..

[31] Golden J.W., Hooper J.W. (2008). Heterogeneity in the A33 protein impacts the cross-protective efficacy of a candidate smallpox DNA vaccine. Virology.

[32] Cohen M.E., Xiao Y., Eisenberg R.J., Cohen G.H., Isaacs S.N. (2011). Antibody against extracellular vaccinia virus (EV) protects mice through complement and Fc receptors. PLoS ONE.

[33] Mucker E.M., Freyn A.W., Bixler S.L., Cizmeci D., Atyeo C., Earl P.L., Natarajan H., Santos G., Frey T.R., Levin R.H. (2024). Comparison of protection against mpox following mRNA or modified vaccinia Ankara vaccination in nonhuman primates. Cell.

