# Supplementary material for: Enhanced Immunogenicity and Affinity with A35R-Fc-Based Chimeric Protein Compared to MPXV A35R Protein
Source: Viruses. 2025 Jan 16;17(1):116. doi: 10.3390/v17010116 (PMC11768982; doi:10.3390/v17010116)
Supplement: Supplementary file 1 [file viruses-17-00116-s001.zip › viruses-3387469-supplementary.pdf]

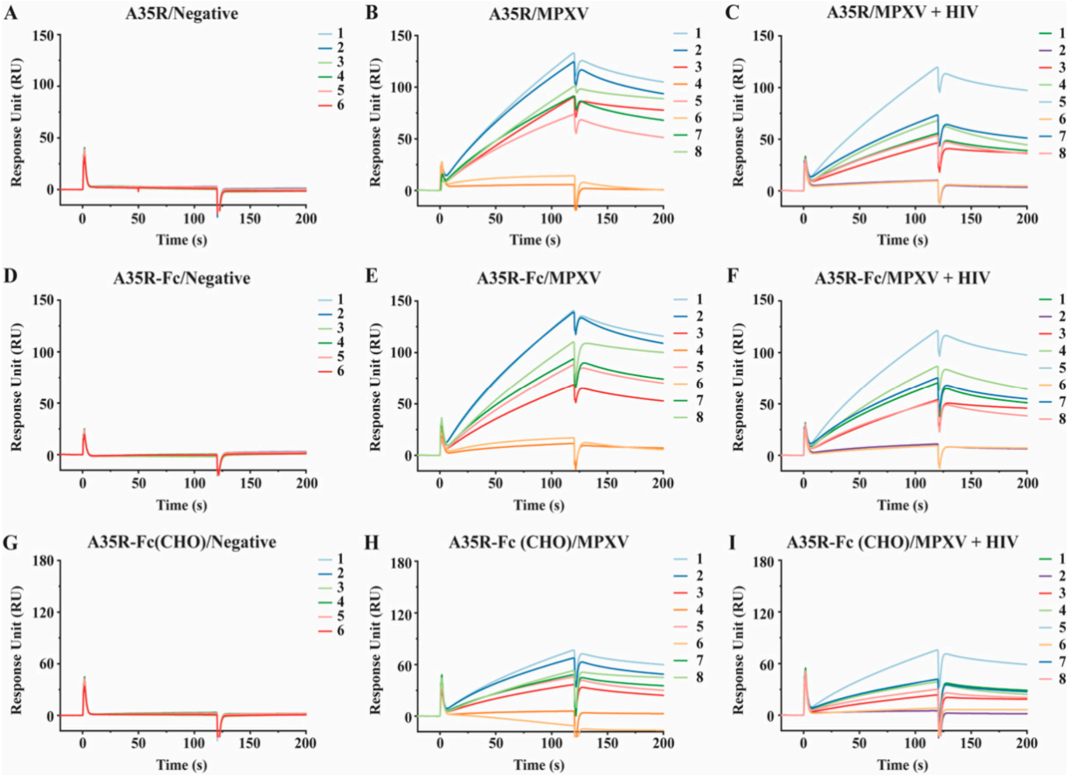


**Figure S1. Binding curves of A35R-FC with the human plasma samples determined by a biolayer interferometry assay.** Data show the change in RU signal over time at a certain dilution (serum dilution). These tests represent three replicates.
